# Improving distribution models of riparian vegetation with mobile laser scanning and hydraulic modelling

**DOI:** 10.1371/journal.pone.0225936

**Published:** 2019-12-05

**Authors:** Tua Nylén, Elina Kasvi, Jouni Salmela, Harri Kaartinen, Antero Kukko, Anttoni Jaakkola, Juha Hyyppä, Petteri Alho

**Affiliations:** 1 Department of Geography and Geology, University of Turku, Turun yliopisto, Finland; 2 Department of Remote Sensing and Photogrammetry, Finnish Geospatial Research institute FGI, National Land Survey of Finland, Masala, Finland; 3 Aalto University, Department of Built Environment, Aalto, Finland; Ghent University, BELGIUM

## Abstract

This study aimed at illustrating how direct measurements, mobile laser scanning and hydraulic modelling can be combined to quantify environmental drivers, improve vegetation models and increase our understanding of vegetation patterns in a sub-arctic river valley. Our results indicate that the resultant vegetation models successfully predict riparian vegetation patterns (Rho = 0.8 for total species richness, AUC = 0.97 for distribution) and highlight differences between eight functional species groups (Rho 0.46–0.84; AUC 0.79–0.93; functional group-specific effects). In our study setting, replacing the laser scanning-based and hydraulic modelling-based variables with a proxy variable elevation did not significantly weaken the models. However, using directly measured and modelled variables allows relating species patterns to e.g. stream power or the length of the flood-free period. Substituting these biologically relevant variables with proxies mask important processes and may reduce the transferability of the results into other sites. At the local scale, the amount of litter is a highly important driver of total species richness, distribution and abundance patterns (relative influences 49, 72 and 83%, respectively) and across all functional groups (13–57%; excluding lichen species richness) in the sub-arctic river valley. Moreover, soil organic matter and soil water content shape vegetation patterns (on average 16 and 7%, respectively). Fluvial disturbance is a key limiting factor only for lichen, bryophyte and dwarf shrub species in this environment (on average 37, 6 and 10%, respectively). Fluvial disturbance intensity is the most important component of disturbance for most functional groups while the length of the disturbance-free period is more relevant for lichens. We conclude that striving for as accurate quantifications of environmental drivers as possible may reveal important processes and functional group differences and help anticipate future changes in vegetation. Mobile laser scanning, high-resolution digital elevation models and hydraulic modelling offer useful methodology for improving correlative vegetation models.

## Introduction

Correlative modelling of vegetation patterns enables the examination of the probable importance of environmental drivers and their effects on vegetation with static survey data (e.g. [1,2]). In addition, the models can be used to project vegetation patterns in future conditions ([1,2]). Recent research highlights the importance of the incorporation of all relevant factors into the models and measuring them at the considered geographical scale (e.g. [3–6]). Moreover, it is important to select biologically appropriate variables as quantifications of those factors. Using such variables in vegetation models improves our ability to draw relevant conclusions on future vegetation patterns and their drivers ([7,8]).

Sub-arctic river valleys are one of the regional hotspots of biodiversity. They are characterized by high geodiversity (variation in topography, geology and earth surface processes) and function as a transition and corridor ecosystem. They therefore provide microhabitats and refuge for tundra species and fluvial specialist species ([9–14]). Moreover, they allow the extension of many boreal species into the (sub-) Arctic as outlier populations (e.g. [15,16]). Through the maintenance of a wide range of microhabitats, the river valleys may be one of the key environments for preservation of biodiversity when the climate changes ([14,17]). To better understand the probable fate of vegetation in the river valleys, it is important to know how vegetation patterns are maintained in current climatic conditions ([18]).

Globally however, sub-arctic river valleys are relatively simple ecosystems with steep environmental gradients. In addition, they are characterized by seasonal flooding (e.g. [19,20]). This intense, regular and predictable disturbance may make them suitable for examining modelling applications (e.g. [21]). In this paper, the term “disturbance” is used on one hand both for events potentially causing loss of biomass and physiological stress, and on the other hand for both infrequent and frequent events related to flooding and animal behaviour (e.g. [22–24]).

A wide variety of direct and indirect factors (c.f. [7]) have been suggested to influence species richness, distribution and abundance, depending strongly on the spatial and temporal dimensions of the study setting. Seven essential factors are generally considered to drive the formation of habitats and vegetation patterns: light, water, nutrients, temperature, biotic interactions, disturbance and the availability of carbon dioxide ([25–27]). At the local scale, the availability of carbon dioxide varies very little and is not considered an important driver of vegetation patterns (e.g. [28–31]).

Light, water and nutrients constitute site productivity. Mainly, increasing site productivity is expected to have a positive effect on the probability of vegetation establishment and primary production ([32,33]). Species richness is expected to peak at a certain productivity level, since beyond that level, the most competitive species are assumed to exclude others (competitive exclusion; [13,33]). Soil pH, organic matter and the amount of litter are commonly accepted proxies for nutrient and long-term soil water conditions, since they either control or are controlled by these drivers ([34,35]).

Disturbance is generally assumed to have a negative effect on species richness, probability of vegetation occurrence and vegetation abundance, by destroying plants and removing seeds ([32]). Only in most productive environments, disturbance is expected to increase species richness ([32,33,36]). In the riparian environment, however, the complexity of earth surface processes and biotic processes has been suggested to create diverging and unpredictable disturbance-diversity relationships (reviewed by [12]). Riparian vegetation is subject to regular flooding, caused by precipitation peaks and snowmelt. In addition, slope processes cause both gradual and catastrophic disturbance events along the banks ([12]). These disturbances are one of the most important factors influencing species richness and distribution in river valleys (e.g. [13,37–39]). Commonly, disturbance is quantified using proxies instead of measuring it directly. Most studies have estimated the net effect of fluvial disturbances in riparian environments using e.g. the elevation relative to low water mark as a surrogate (e.g. review by [12]). However, disturbance can be directly quantified by measuring erosion and accumulation, unit stream power ([40], as a driver of vegetation patterns [41]), analysing river bank stability ([42]), animal paths and burrows or the frequency and duration of flooding ([43]).

Biotic interactions have also been shown to be important in maintaining vegetation patterns. Theory and studies in disturbance-dominated environments indicate that disturbance efficiently reduces competition (e.g. [36,43–45]) but does not remove it completely ([12]). *Empetrum nigrum* is a widespread generalist and competitive species, and its presence has been shown to have a strong negative effect on majority of vascular plants of the sub-arctic environment ([46]). In turn, vegetation is expected to have a notable effect on the mobility of sediment, therefore influencing the intensity of earth surface processes (e.g. [42,47]). This stabilizing effect of established vegetation is expected to facilitate other species (nurse plant effect; [44,48]).

The responses of species groups to environmental drivers are known to diverge due to different adaptation strategies ([14,49]). The lower boundary of species ranges along river banks are expected to correspond to species’ sensitivity to flooding ([50–52]). Many plants of the riparian environment have adaptations to submergence and the movement of water and sediment ([12,52]) giving them the opportunity to establish in highly dynamic but competition-free sites.

Mobile laser scanning can be cost-efficiently implemented in narrow corridor-type ecosystems to produce (potentially a series of) high-resolution digital elevation models (DEM; e.g. [53–55]). Consecutive DEMs allow precise calculation of erosion and accumulation along river banks ([56]). When the DEMs are combined with *in situ* measurements of water flow conditions in a few locations, hydraulic modelling can be utilised to calculate a time series of 3D flow fields for the river valley (e.g. [57,58]), and further transformed into e.g. unit stream power and frequency and duration of flooding. However, to our knowledge the laser-scanning based quantification of erosion and accumulation or hydraulic variables have not been utilized in improving correlative modelling of vegetation patterns.

Using elevation as a proxy variable for multiple disturbance gradients also helps to alleviate issues of multicollinearity when there are several parallel gradients. However, multicollinearity issues can be managed with variable selection prior to statistical multivariate modelling and using advanced modelling methods that are less sensitive to multicollinearity issues (e.g. boosted regression trees; [59,60]). These methods have potential in clarifying the relationship between vegetation patterns, disturbance and other drivers ([61,62]).

This study aims to 1) examine how correlative models of local-scale vegetation patterns in riparian ecosystems can benefit from incorporating both directly measured environmental variables and variables based on laser scanning and hydraulic modelling (c.f. [6]). The outcomes of the vegetation models are utilized to examine, 2) what are the most influential factors in determining vegetation patterns of the whole community and across functional groups in this study setting and 3) which components of disturbance are most influential for vegetation patterns and how they limit vegetation establishment? This is achieved by combining high-resolution survey data of e.g. light and soil conditions to accurate estimates of disturbance patterns. Disturbance is determined with modern laser scanning techniques (erosion and accumulation) and hydraulic modelling (fluvial disturbance variables). Moreover, advanced statistical modelling methods are applied to draw robust conclusions from the data. The study is conducted in a sub-arctic river valley. This is a relatively simple system with few interacting species and steep environmental gradients ([19,20]). Biotic interactions and the influence of abiotic drivers are expected to be easily detected in such a setting ([21,36]). The study area may therefore be suitable for examining the modelling approach.

## Study area

**Fig 1:**
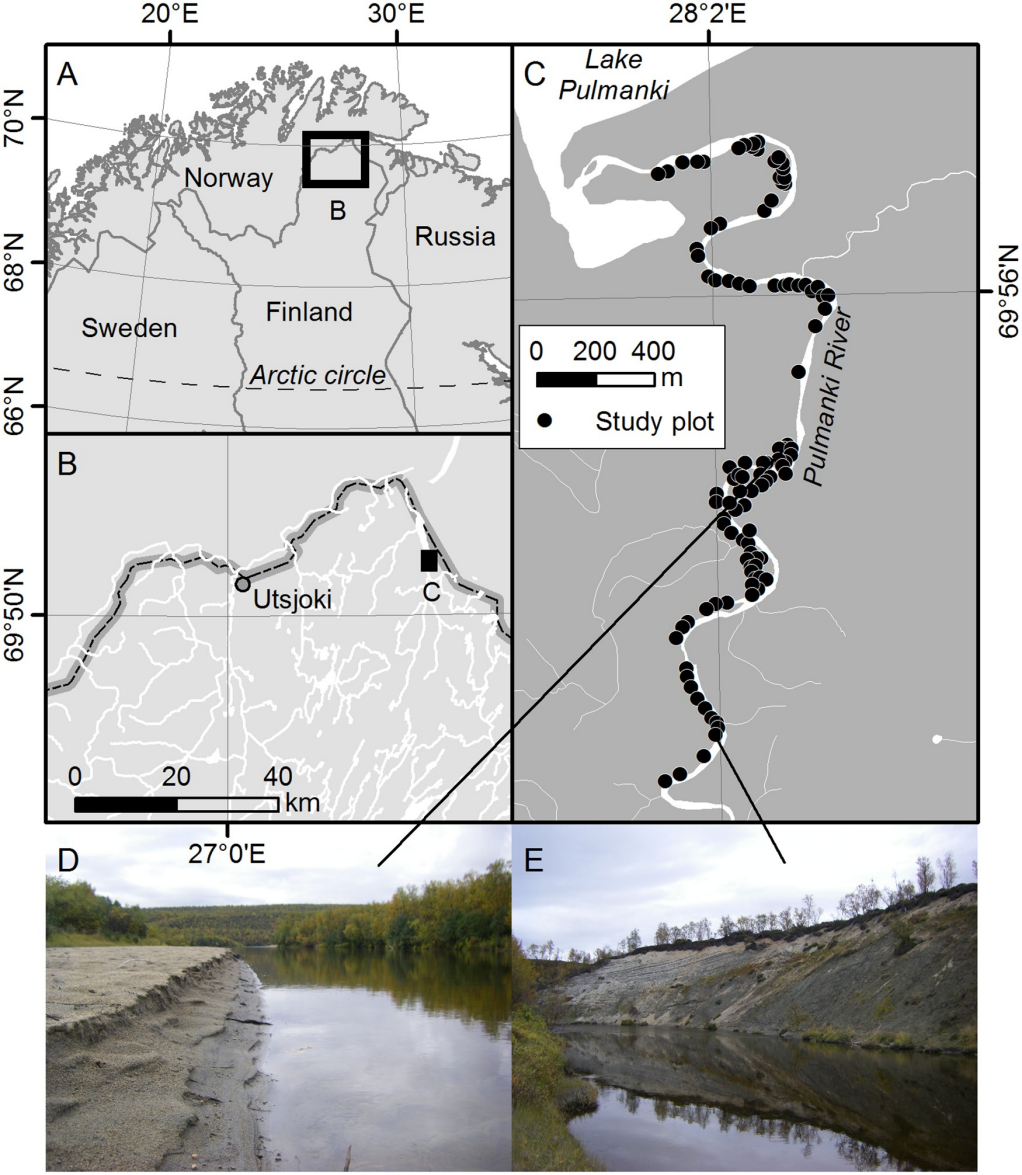
Study area. Location of the study area (Lower Pulmanki River; N 69° 55.111', E 028° 01.664'; N 69° 56.281', E 028° 02.631') and surveyed plots in northernmost Finland (A-C) and representative scenery from the area (D-E) (Background data: Topographic database 2015, General map 1:1 000 000 2015 and Elevation model 10 m 2015 of the National Land Survey of Finland).

The study area was located in the northernmost Finland, and constituted a 3.5-km reach of the lower Pulmanki River (Fig 1; Lower Pulmanki River; N 69° 55.111', E 028° 01.664'; N 69° 56.281', E 028° 02.631'). Before entering into Pulmanki Lake, the sub-arctic river flows along a 1 km-wide depression filled with glaciofluvial sand and gravel deposits ([19]; Finnish Geological Survey Surficial deposits database). The river valley is characterised by distinct annual variation in water level with one high snowmelt-induced spring flood ([55]). The interannual variation in the water surface elevation of the spring flood is distinct ([55]). This creates a steep environmental gradient from the highly dynamic river bed to relatively stable river banks. The Lake Pulmanki may cause a backwater effect during the spring flood. Due to annual differences in the strength of the backwater effect, the water surface slope varies notably depending from year to year.

The study area is located at the transition zone between boreal taiga and arctic tundra ([63]). The vegetation is mainly influenced by the northern location, proximity to the Arctic Ocean (Barents Sea) and the fluvial landscape. The river valley is surrounded by mountain birch (*Betula pubescens* ssp. *czerepanovii*) forests that extend close to the waterline at sheltered locations. Alpine dwarf shrub heaths dominate the higher terraces and fen vegetation covers local depressions. The dynamic sand bars and steepest slopes remain sparsely vegetated (Fig 1).

The study considered the entire channel and floodplain along the 3.5-km stretch. The study area was determined by bankfull flood extent and covered a total of 27.5 ha. Permanently submerged parts of the channel were excluded from the analysis. This lower margin of the study area followed the river’s shoreline in low discharge conditions in September 2016, at c. 15 and 14 meters above sea level upstream and downstream, respectively. The upper margin of the study area was determined from aerial photography from high flow conditions of spring 2013, when the water level had reached a typical spring maximum ([20]). An extra ten meters outside the 2013 flood extent was added to the study area to cover the entire flood frequency gradient.

## Methods

### General study design

To address the study questions, 1) we built complex statistical multivariate models to examine if the incorporation of biologically meaningful environmental variables based on laser scanning and hydraulic modelling (“full” models) significantly improved vegetation models. They were compared to models were plot elevation was used as a proxy for the multiple environmental factors (“simple models”; following the methodology of [64]). 2) We then analysed the importance of each factor in the “full” models in explaining the vegetation patterns in this study setting. The effects of the most influential factors on vegetation patterns were examined. 3) Finally, we examined the relative influence of the disturbance components and their effects on vegetation patterns in more detail.

We used a correlative multivariate modelling technique to examine the influence of environmental factors on the richness, distribution and abundance of vascular plants and cryptogams of the lower Pulmanki river below the canopy layer. We considered biotic interaction, disturbance and site productivity variables in the models. First, we analysed their effects on the total number of species and the total distribution and abundance of vegetation. Second, the analyses were repeated for six functional species groups. For biotic interactions, we accounted for the shading effect of the canopy and the influence of four dominant species. We considered disturbance in the form of bioturbation, erosion, sediment accumulation, flooding and stream power. Site productivity was quantified as solar radiation, organic litter availability, soil pH, soil water content and soil organic matter. The input data for the statistical analyses is provided as supporting information (S1 Table) and described in detail in the following sections.

### Field visits and study plots

The data were collected in the lower Pulmanki river valley during eight consecutive years, between 2009 and 2016. River flow conditions were measured during the spring flood period each year. The elevation and bathymetry of the lower Pulmanki river valley were measured in autumn 2014 and again in autumn 2016. Simultaneously in autumn 2016, vegetation, soil and solar radiation data were collected in 106 study plots.

The water flow conditions of the study reach were measured each year (2009–2016): discharge was measured daily during each spring flood peak with an Acoustic Doppler Current Profiler (ADCP; SonTek RiverSurveyor M9 0.5 MHz equipped with an echo sounder with 1% accuracy). Two water pressure sensors (Solinst Levelogger Gold model 3001 with a 15 minute logging interval and 0.05% accuracy) were installed in the river bed to measure water level variation. One water pressure sensor was at a mid-reach location and the other was located at the downstream boundary. The water levels were recorded from early May to early September each year.

The topography of the dry parts of the study area was measured twice with a backpack mobile laser scanner (updated versions of [65]; Table 1), in September 2014 and in September 2016. The laser scanner, survey grade RTK-GNSS receiver, inertia measurement unit, batteries and a handheld survey laptop were attached to a backpack system that was carried by one person. Survey paths in the field were designed to enable a multiangular survey campaign in order to avoid extreme oblique angles (> 80 degrees from the ground) and shadow effects of the river banks and trees. The backpack system was carried mostly by walking along the river banks and on the point bars. One additional survey path was followed with the backpack system placed on an inflatable boat and navigating the boat down the river. Target spheres (26 targets in 2014 and 36 targets in 2016) were used to verify the georeferencing and rectify the tilting of the point cloud. Laser scanning produced three-dimensional point clouds of the study area with an average horizontal point density of 2000–40000 points per m^2^ depending on the range from the trajectory. Point density was highest in the central parts of the study area while highest flood banks remained out of reach of the scanner. Simultaneously, the bathymetry of the river was measured with the ADCP. The topographic, bathymetric and water flow data were later used as inputs for digital elevation models and hydraulic modelling.

**Table 1:** Systems used for backpack mobile laser scanning.

	2014	2016
**Laser scanner**	Faro Focus S120 Phase shift ranging	RIEGL Vux-1HA Time-of-Flight ranging, multiecho
**Laser wavelength**	905 nm	1550 nm
**Positioning system**	NovAtel Flexpak 6 GNSS receiver, Pinwheel 702-GG GNSS antenna, UIMU-LCI inertial unit	

In total 200 coordinate points were randomized inside the study area (with ArcGIS Create Random Points tool within the study area polygon). In early September 2016, 106 of these coordinate points (92 points along the western side of the river and 14 points covering the largest sand bar on the eastern side of the river; remaining 94 random points on the eastern side were not visited due to time constraints) were navigated to in the field using a handheld Garmin GPS receiver and study plots were established there (Fig 1). During these low discharge conditions, the entire gradient from dynamic sand bars to rarely submerged banks was dry. Of the surveyed plots, 34 (32%) had not been inundated by the river during the observed period (2009–2016) while 56 (53%) had experienced flooding every spring.

The accurate plot locations were measured with a survey grade RTK-GNSS (Real Time Kinematic Global Navigation Satellite System; Trimble R10 with 5 cm horizontal accuracy) and photographed once. Plots of 1 m^2^ were temporarily marked on the ground, their vegetation (see following chapter for details) and litter layer (litter cover and thickness) inventoried and soil cores collected. Soil samples of c. 2 dl were collected just below the litter layer, stored and brought to laboratory following standard procedures (ISO 11464). Four of the 106 samples were lost or destroyed in the process. Photosynthetically Active Radiation (PAR) was measured once (as a five-minute average) at each plot location above the canopy and below the shrub layer with a LI-COR LI-190R Quantum Sensor. The measurements were made in uniformly cloudy conditions but during five consecutive days and different times of the day (between 10 am and 7 pm).

No permissions were required for the study in the lower Pulmanki River (mostly privately owned), based on the Finnish law on everyman’s rights. The field sampling did not involve endangered or protected species.

### Vegetation inventory and variables

During the field visit in early September 2016, the number of species (**species richness**; Table 2), cover and maximum height of the vegetation and of individual functional groups were measured in the field, once in each of the 106 study plots. Functional groups of lichens, bryophytes, graminoids, forbs, dwarf shrubs and shrubs were considered. Pteridophytes were included in variables describing the total vegetation cover (total species richness, total species distribution, total abundance) but not considered individually. In addition, the cover and maximum height of four common species of the shrub and herbaceous layers (*Empetrum nigrum*, *Vaccinium vitis-idaea*, *Vaccinium uliginosum*, *Juniperus communis*; present in > 10% of the plots) were measured. These species have also been shown to have strong impact of other species of the sub-arctic vegetation (e.g. [46,64]).

**Table 2:** Descriptive statistics of the variables.

Variable	Dimension	Class	N	Min	Median	Mean	Max	Sd
**Species richness**								
Total	count	numeric	106	0	7	6.4	18	4.6
Lichen	count	numeric	106	0	0	0.5	6	1.2
Bryophyte	count	numeric	106	0	2	1.8	6	1.6
Graminoid	count	numeric	106	0	1	1	4	0.8
Forb	count	numeric	106	0	0	1	6	1.4
Dwarf shrub	count	numeric	106	0	1	1.2	4	1.2
Shrub	count	numeric	106	0	0.5	0.7	6	0.9
**Species distribution**								
Total	binomial	numeric	106	0	1	0.8	1	0.4
Lichen	binomial	numeric	106	0	0	0.2	1	0.4
Bryophyte	binomial	numeric	106	0	1	0.7	1	0.5
Graminoid	binomial	numeric	106	0	1	0.7	1	0.4
Forb	binomial	numeric	106	0	0	0.4	1	0.5
Dwarf shrub	binomial	numeric	106	0	1	0.6	1	0.5
Shrub	binomial	numeric	106	0	0.5	0.5	1	0.5
**Abundance (vegetation volume)**								
Total	dm^3^	numeric	106	0	197.1	281	997.6	280.1
Lichen	dm^3^	numeric	106	0	0	1.2	24	3.8
Bryophyte	dm^3^	numeric	106	0	2	14.5	100	24
Graminoid	dm^3^	numeric	106	0	8.1	75.4	644	148.3
Forb	dm^3^	numeric	106	0	0	21	432	57.2
Dwarf shrub	dm^3^	numeric	106	0	0.3	88.7	504	132.4
Shrub	dm^3^	numeric	106	0	0	60	819	128.4
**Biotic interaction**								
Light attenuation	%	numeric	106	-31.4	12.1	20.1	88.6	26.4
*Empetrum nigrum*	dm^3^	numeric	106	0	0	15.6	189	34.6
*Vaccinium vitis-idaea*	dm^3^	numeric	106	0	0	25.1	161	41
*Vaccinium uliginosum*	dm^3^	numeric	106	0	0	48.9	504	105
*Juniperus communis*	dm^3^	numeric	106	0	0	17.3	819	88.7
**Disturbance**								
Bioturbation	binomial	numeric	106	0	0	0.2	1	0.4
Erosion/accumul.*	dm^3^	numeric	106 (62)	-333.7	0	-5.2	293.8	86.5
Peak water depth 2016**	m	numeric	106	0	0	0.3	1.5	0.4
Peak water depth 2009–2016**	m	numeric	106	0	1.1	1.1	3.1	1
Peak unit stream power 2016**	W m^-2^	numeric	106	0	0	0.3	2.5	0.5
Peak unit stream power 2009–2016**	W m^-2^	numeric	106	0	2.2	3.3	15.9	4
Time since flood**	years	numeric	106	0	0	3.2	8	3.7
**Site productivity**								
Solar radiation***	Wh m^-2^	numeric	106	247336	476908	457787	606927	76921
Litter volume	dm^3^	numeric	106	0	10	29.9	200	46.4
Soil pH		numeric	102	4.9	5.8	5.8	6.4	0.3
Soil water content	%	numeric	102	0.8	15.7	18.3	65.8	14.2
Soil organic matter	%	numeric	102	0.2	1	2.9	20.2	4.2
Distance along river	m	numeric	106	104	1733	1996	4016	1078

The erosion/accumulation variable (*) has been calculated based on two consecutive DEMs (n = 62; see chapter Disturbance for details) and assumed to be zero for plots without consistent DEM coverage (n = 44).

The variables marked with an asterisk (**) are based on hydraulic modelling (see Hydraulic modelling).

The solar radiation variable (***) has been modelled based on the 2016 DEM (see Soil samples and site productivity). All these variables have then been extracted from the model results for each of the 106 plot locations.

In total, all analyses were run with 102 complete observations. The remaining four observations lacked measurements of one or more variables and where thus excluded from modelling.

Cover values were converted into binomial **species distribution** variables (Table 2). Aboveground volume (cover * height) of each group or species was used as a proxy for aboveground **abundance** of the group (Table 2). Total aboveground vegetation abundance was estimated as the sum of values of the six functional groups (Table 2).

### Biotic interaction variables

**Light attenuation** (Table 2) below the shrub layer was calculated from the measurements of PAR above the canopy and below the shrub layer as:

$$Light\;attenuation\;=\;\frac{\left({PAR}_{above\;canopy}-{PAR}_{below\;shrub\;layer}\right)}{{PAR}_{above\;canopy}}*100\%$$ (1)

The sensitiveness of the light attenuation variable to diurnal effects (e.g. [66]) was analysed with linear regression analysis (light attenuation as the function of measurement time). No linear, polynomial or sinusoidal model significantly reduced the residual deviance (p > 0.01 for F-statistic). We used ANOVA to assess whether there were differences between the five different measurement days (potentially due to different weather condition; [66]). There were no significant differences between the measurement days. Based on these results, no adjustments were made to the light attenuation variable. The volume of the four **dominant species** was used as a proxy for the intensity of species interactions ([64]; Table 2).

### Disturbance variables

The surface area of reindeer, lemming and human paths was measured and the number of animal burrows calculated in each study plot in the field. Due to small variation (large majority of plots had no clear paths or burrows), these numbers were converted into a binomial **bioturbation** variable (present = 1/absent = 0; present in 29% of the plots; Table 2).

The elevation point data for 2014 and 2016 were filtered by removing points that had an intensity value less than a defined threshold. This removed points from the air and below ground, as well as some real hits from the targets far from the scanner or with low reflectivity. Air points were deleted by computing the number of points within a certain radius in the air and removing the points if the density was less than the threshold. The system-specific thresholds applied in our study were 500 (intensity threshold, scale 0–2044) and 10 pts within a 50 cm radius (cf. [67]). Subsequently, vegetation was classified out with an algorithm provided by TerraScan software, and only the ground points were used in further analyses. After the filtering procedure, the data (Table 1) were resampled into regular point clouds describing the topography of the dry areas with 50 cm point spacing. Point clouds were registered to EUREF-FIN coordinate system and N2000 height system. Two triangulated irregular networks (TIN, for 2014 and 2016) were calculated from the resampled point elevation data and rasterized into digital elevation models (DEM) with a horizontal resolution of 50 cm.

The RTK-coordinates of the study plots were used to tie field observations to the DEMs. Of the 106 study plots, 62 plots had consistent DEM coverage for 2014 and 2016. **Erosion or accumulation** at the location of a study plot was calculated by subtracting the 2014 elevation from the 2016 elevation value and multiplying it with the plot surface area (1 m^2^; Table 2). Study plots located in the highest flood banks and most sheltered places (44 plots) were masked from one or both DEMs. They were assumed to have experienced no erosion or accumulation during the two years.

#### Hydraulic modelling

Hydraulic modelling was used to determine the inundation area, and the spatial variability of water depth and stream power during the flood peak (maximum discharge) of each year during 2009–2016. Hydraulic model resolves the fluid motion in each grid cell over a series of boundary conditions. In this study, a steady state model (constant flow situation) was run for each year (in total eight runs; Table 3).

**Table 3:** Boundary conditions and calibration values for hydraulic modelling.

Run	Date	BC 1 (m^3^s^-1^)	BC 2 (m)	Calibration WL, measured (m)	Calibration WL, modelled (m)	Manning’s n	Eddy viscosity	Slope (m/m)
1	15.5.2009	41	15.87	15.94	15.94	0.016	0.55	0.000241
2	21.5.2010	49	16.64	16.68	16.67	0.016	0.55	0.000558
3	17.5.2011	22	14.27	14.90	14.89	0.012	0.55	0.000674
4	21.5.2012	41	16.05	16.13	16.12	0.019	0.58	0.000214
5	19.5.2013	65	15.30	15.70	15.71	0.016	0.55	0.000598
6	23.5.2014	44	14.87	15.23	15.24	0.009	0.55	0.000576
7	26.5.2015	30	15.15	15.51	15.52	0.028	0.70	0.000527
8	17.5.2016	15	15.02	15.19	15.18	0.026	0.55	0.000362

Boundary conditions and calibration values used for each hydraulic modelling run, representing yearly flood peaks (maximum discharge) in 2009–2016. Date = timing of the yearly flood peak; BC 1 = discharge, upstream boundary condition; BC 2 = water surface elevation, downstream boundary condition, WL = water level.

The model geometry was based on field measurements of bathymetry and topography of the channel edges in September 2016. The bathymetric ADCP data and the laser scanning-based elevation data for dry areas were combined in a GIS software into a seamless river geometry. The geometry was interpolated into a raster grid (cell size 1.0 m). The same geometry was used in each model run.

A curvilinear grid representing the study reach was created in Delft3D software. Compared to a rectangular grid, the curvilinear grid allows for finer grid resolution over the areas of interest and more accurate simulation of the processes along the river boundaries and bends. The grid cell size varied between 3 and 8 meters in the channel area, depending on channel geometry. Thus, the resolution of the geometric sample data set was higher than the grid resolution, and the average value of the sample points falling inside a grid cell was used.

Discharge (BC 1 in Table 3) and water surface elevation (BC 2 in Table 3) of the peak flood event were used as the upstream boundary and downstream boundary conditions, respectively. The discharges were based on the ADCP measurements and the highest discharge of each year was used as the boundary value. The measurements of the two water pressure sensors were transformed into water level changes at the mid-reach and downstream boundary and tied to geographic coordinates using the RTK-GNSS. The water surface elevation of the peak discharge event of each year was used as the downstream boundary value in the hydraulic modelling. The water level at the mid-reach location was used to calibrate the model.

In the modelling process, the two-dimensional Reynolds averaged momentum and continuity equations were implemented on a curvilinear, unstructured grid. The peak flood event of each year was modelled separately as a steady state model and the manning’s friction and horizontal eddy viscosity coefficients were adjusted so that the mid-reach water level corresponded exactly to the measured mid-reach water level (Table 3). A maximum difference in the modelled and measured water levels of 2 cm was accepted. A uniform friction value was used over the modelling area. The governing equations are described in detail in ([68]). A modelling period of 24 hours was used to make sure that the flow had enough time to stabilise during the simulation. A time step of five minutes was used, and it took approximately 18 time steps for the model to stabilise. The results of time step 100 were extracted to the results of the hydraulic modelling.

The spatial distribution of water depth and flow velocity of each run was extracted over the entire study area. In addition, the spatial distribution of unit stream power was calculated from the modelling results. The unit stream power (W m^-2^) is the rate of energy dissipation of water against the channel bed per unit area and is calculated by ([40]):

ω=ρgDSv (2)

where ρ is the density of water (kg m^-3^), g is the gravitational acceleration (m s^-2^), D is the flow depth (m), S is the water surface slope and v is the flow velocity (m s^-1^). The water surface slope of the flood event was calculated between the simulated water levels at the upstream and downstream of the study reach. Unit stream power has been widely used by researchers to quantify energy dissipation of flow and is strongly related to the capacity of the river to transport sediment ([69]).

Water depth and unit stream power were extracted for each study plot from the hydraulic modelling results. They were extracted separately for the vegetation survey year 2016 (representing **peak water depth** and **peak unit stream power in 2016**) and the entire period 2009–2016 (**peak water depth** and **peak unit stream power in 2009–2016**), to account for short-term and long-term patterns, respectively (Table 2). In addition, **time since flood** (since last inundation; [33]) was calculated for each plot (Table 2).

### Soil samples and site productivity

The 2016 DEM was further used to calculate solar radiation, i.e. global insolation, in each plot. Before calculation, the DEM was extended outwards from the study area using the coarse contour and point elevation data of the National Land Survey of Finland (Topographic database, version 2016). This enabled taking into account the shadow effects of nearby fells. The point elevations (resampled elevation point cloud and Topographic database) and contours were used to calculate a topographic TIN model. The TIN model was then rasterized into 50 cm horizontal resolution. **Solar radiation** was calculated for the plot coordinates, accounting for latitude, seasonality and daily variation, elevation, slope, aspect and shadows cast by surrounding topographic features (Table 2; [70]). It was calculated with the Solar Radiation toolset of ArcGIS Spatial Analyst ([71]).

Cover and thickness of the litter layer, measured in the field in September 2016, were converted to **litter volume** (Table 2). The 102 soil samples from September 2016 were analysed in the laboratory following standard procedures for **soil organic matter** (SFS 3008), **soil water content** (SFS 3008) and **soil pH** (ISO 10390; Table 2).

### Variable selection

Preliminary selection resulted in 17 potential predictor variables: five biotic, seven disturbance and five site productivity variables. To reveal possible collinearity issues (potentially caused by underlying causal relationships or artefacts of data collection) between these variables, the Spearman rank correlation (rho) was calculated between all 17 variables and all variable pairs with high correlations (|rho| ≥ 0.7) were examined in detail (S2 Table).

The variables based on hydraulic modelling were interlinked to the degree that only one of them was used in further analyses. The potentially best fluvial disturbance predictor was selected prior to the multivariate modelling, individually for each response variable. It was selected using Spearman rank correlation as an indicator of predictive power.

Four high predictor correlations remained in the final set of predictor variables. Litter volume and soil organic matter, as well as soil organic matter and water content were strongly correlated. The abundance of *Vaccinium vitis-idaea* was strongly correlated with litter volume and soil organic matter. These predictor variables were included in the analyses since they were expected to be biologically meaningful and were quantified by independent measurements. However, potential collinearity issues were taken into account when interpreting the results.

### Statistical modelling

For all 21 response variables, we fitted two statistical model variants: a “full” model including variables based on laser scanning and hydraulic modelling, and a “simple” model were these variables were substituted by a single elevation variable. The common part of the “full” and “simple” models for all response variables was:

“Full”model:responsevariable~lightattenuation+bioturbation+erosion/accumulation+fluvialdisturbancevariable+solarradiation+littervolume+soilpH+soilwatercontent+soilorganicmatter (3)

where the fluvial disturbance variable was selected individually for each response variable (S3 Table).

“Simple”model:responsevariable~lightattenuation+bioturbation+elevation+littervolume+soilpH+soilwatercontent+soilorganicmatter (4)

In addition, the abundance of the four dominant species was included in the lichen, bryophyte, graminoid and forb models. The abundance of *Juniperus communis* (a shrub species) was included in the dwarf shrub models and the abundances of the three dominant dwarf shrub species (*Empetrum nigrum*, *Vaccinium vitis-idaea* and *Vaccinium uliginosum*) were included in the shrub models. The modelling was repeated for such “total” vegetation variables that excluded the four dominant species. This enabled the examination of the influence of dominant species on the remaining vegetation.

Boosted regression tree (BRT) method was utilised to fit the multivariate models ([59]). BRT is an ensemble machine learning technique that estimates the relationship between a response variable and a set of potential predictor variables without *a priori* specification of the data model ([59]). Species richness was modelled using a Poisson distribution of errors and logarithmic link function. Species distribution variables were modelled with a Bernoulli distribution and logit link function. Logarithmic transformation (with Gaussian distribution of errors) was applied to the non-normally distributed abundance variables, to linearize the models. Up to three-way interactions between predictor variables were modelled (interaction depth = 3) and other model settings were kept to defaults (learning rate 0.001, step size 50, bag fraction 0.5). The optimum model (optimal number of iterations) was determined with ten-fold cross-validation with random assignment ([72]).

Model performance was evaluated with five-fold cross-validation with random assignment ([72]). We compared observed values against predicted values for the validation data of each cross-validation fold. Species richness and abundance models were evaluated by calculating Spearman rank correlations of observed and predicted values. For occurrence predictions, area under the curve of a receiver operating characteristic plot (AUC; [72]) was calculated. Predicted occurrence probabilities were first converted to binary presence/absence data using a species-specific threshold maximizing sensitivity and specificity of the model (for details see [73]). Performance of the “simple” and “full” models were compared with a paired two-tailed Z test ([74]).

The relative influence of predictor variables in “full” models was determined with Friedman’s ([75]) method (based on the reduction of squared error attributable to each variable, averaged over all trees and normalised so that the sum of the predictors’ relative influences was 100). The effects of the most influential predictor variables (with relative influence > 7% in models of total species richness, distribution and abundance, and relative influence > 10% in the functional group models) on vegetation variables in each model were plotted (after integrating out the effects of all other variables; [75]) and visually classified into four categories: positive, negative, unimodal and U-shaped.

The BRT model residuals were examined for spatial autocorrelation by calculating Moran’s I for discrete distance classes using a lag of 10 m and testing for significance ([76]). The residuals of six models showed significant spatial autocorrelation, indicating that there was spatial dependence of the observations that could not be accounted for by the fitted model (S4 Table). Therefore, the spatial structure of the data was summarized into a dummy variable describing the position of plots along the river channel (distance from the upstream edge of the study area along main flow path). In addition, this variable reflected the potential dispersal and other effects along the course of the river ([18]). This dummy variable was integrated into the BRT models and its incorporation reduced spatial autocorrelation of five model residuals. However, the residuals of the lichen richness model remained spatially autocorrelated (S4 Table), which should be taken into account when examining the results of this model.

The analyses were repeated with generalised linear models ([77]), generalised additive models ([78]) and geographically weighted regression ([79]). The results of the additional analyses were well in line with the main BRT analyses, suggesting that the results were independent of the selected analysis method. Finally, the analyses were repeated excluding litter volume from the predictor variable set. This was done to investigate if important relationships were hidden in the “full” models because of the strong and complex (potential difficulty to separate cause and effect) relationship between vegetation variables and litter volume. However, these additional results were well in line with the main BRT analyses (non-significant to significant reduction in predictive power associated with the exclusion of litter volume from the models and identical rankings of predictor variables based on variable importance across all models).

All statistical analyses were conducted in the statistical software R ([80]). The BRT models were fitted and examined with R packages *dismo* ([81]) and *gbm* ([82]). Spatial autocorrelation was calculated with package *ape* ([83]), distances along the river with package *riverdist* ([84]) and r test with package *psych* ([85]). The additional analyses were conducted with packages *mgcv* ([86]) and *spgwr* ([87]).

## Results

### Influence of mobile laser scanning and hydraulic modelling variables on distribution models

Based on cross-validation, the multivariate BRT models succeeded in predicting total species richness, distribution and abundance of the vegetation relatively well (Rho ≈ 0.8, AUC > 0.96; Table 4). Graminoid and dwarf shrub models performed equally well (Rho > 0.7, AUC > 0.9; Table 4). Generally, lichen, forb and shrub models performed worst (Rho < 0.6, AUC < 0.9; Table 4). Substituting elevation (“simple” models) with biologically relevant variables based on laser scanning and hydraulic modelling (“full” models including erosion and accumulation, fluvial disturbance variables and solar radiation) had no significant influence on model performance (Table 4). This was mainly due to the fact that in this dataset, site productivity variables dominated all models. Neither elevation in “simple” models nor the additional variables in “full” models had notable influence in the models.

**Table 4:** Model evaluation statistics for “simple” and “full” models.

	Species richness				Species distribution				Abundance			
	Simple	Full			Simple	Full			Simple	Full		
Group / species	Rho	Rho	Z	p(Z)	AUC	AUC	Z	p(Z)	Rho	Rho	Z	p(Z)
Total	0.77	0.76	0.10	0.92	0.97	0.97	0.91	0.36	0.82	0.82	0.01	0.99
Total—domin.	0.78	0.78	< 0.01	> 0.99	0.97	0.96	1.34	0.18	0.80	0.80	0.01	0.99
Lichen	0.44	0.49	0.18	0.86	0.81	0.84	0.59	0.56	0.54	0.56	0.26	0.79
Bryophyte	0.58	0.59	0.06	0.96	0.90	0.92	0.89	0.37	0.76	0.78	0.38	0.70
Graminoid	0.68	0.72	0.57	0.57	0.93	0.93	0.45	0.66	0.81	0.84	0.62	0.54
Forb	0.47	0.46	0.64	0.52	0.82	0.79	0.54	0.59	0.51	0.50	0.04	0.97
Dwarf shrub	0.81	0.81	< 0.01	> 0.99	0.90	0.93	1.43	0.15	0.76	0.79	0.56	0.57
Shrub	0.55	0.55	0.33	0.74	0.85	0.85	0.17	0.86	0.59	0.59	0.04	0.97

BRT model evaluation statistics based on five-fold cross-validation. For species richness and abundance variables, Spearman rank correlation (Rho) between observed and predicted values of the validation data is reported. For species distribution variables, area under the curve of a receiver operating characteristic plot (AUC; [72]) values are presented. The statistics are reported for “simple” and “full” models and compared using Z statistic ([74]) and associated p-value. Total—domin. = vegetation variables that exclude four dominant species.

### Most influential factors in determining vegetation patterns

The following results are reported for the “full” models that include variables based on laser scanning and hydraulic modelling. When examining the entire vegetation community, site productivity variables dominated the models. The most influential variable in the models was litter volume, followed by soil organic matter (relative influence 49–83% and 7–21%, respectively; Table 5). Soil water content was influential in the species richness and distribution models (c. 7%; Table 5). Biotic interaction and disturbance variables had generally very little influence in these models (Table 5).

**Table 5:** Relative influences of predictors in the “full” models of total species richness, distribution and abundance.

Type	Species richness		Species distribution		Abundance	
Variable	Total	Total—domin.	Total	Total—domin.	Total	Total—domin.
Light attenuation	6.7	5.5	2.4	2.5	2.0	6.4
*E*. *nigrum*		0.4		0.0		2.0
*V*. *vitis-idaea*		3.6		0.0		1.7
*V*. *uliginosum*		1.7		0.0		0.7
*J*. *communis*		0.0		0.0		0.0
Bioturbation	0.2	0.1	0.1	0.1	0.0	0.0
Erosion/accum.	1.2	0.9	0.9	0.9	0.1	0.2
Fluvial disturbance	0.4	0.4	4.0	4.0	0.1	0.1
Solar rad.	5.2	4.7	1.8	1.9	0.4	1.9
Litter V	**49.5**	**44.8**	**71.5**	**70.8**	**82.6**	**66.1**
pH	5.2	6.3	1.4	1.4	0.9	**8.0**
SWC	**7.0**	6.9	**7.3**	**7.4**	1.2	2.0
SOM	**21.0**	**21.4**	**7.4**	**7.5**	**11.9**	**8.5**
Distance along river	3.5	3.2	3.2	3.3	0.7	2.4
SUM	100.0	100.0	100.0	100.0	100.0	100.0

Relative influence (%; [75]) of predictor variables in the BRT models for total species richness, distribution and abundance. The most influential variables for each response variable (with relative influence > 7%) are highlighted with bold font (see Table 7 for their partial effects). Total—domin. = vegetation variables that exclude four dominant species; Litter V = litter volume; pH = soil pH; SWC = soil water content; SOM = soil organic matter.

Litter volume and soil organic matter were almost invariably the two most important predictor variables in the functional group models (13–57% and 8–55%, respectively, Table 6). Lichen species richness was the only variable not significantly influenced by litter volume or soil organic matter (Table 6). The influence of following biotic interaction variables were highlighted for specific functional groups: light attenuation for the abundance of shrubs, the cover of *Empetrum nigrum* for lichens and the abundances of *Vaccinium uliginosum* and *V*. *vitis-idaea* for the abundances of bryophytes and graminoids, respectively. In addition, fluvial disturbance was influential for lichens and dwarf shrubs, solar radiation for bryophytes and soil pH for lichens, forbs and shrubs (Table 6).

**Table 6:** Relative influences of predictors in the “full” functional group models.

Type	Species richness					
Variable	Lichen	Bryophyte	Graminoid	Forb	Dwarf shrub	Shrub
Light attenuation	0.6	5.2	9.5	5.2	0.8	4.4
*E*. *nigrum*	**18.2**	0.6	0.3	0.2		0.5
*V*. *vitis-idaea*	1.0	7.4	3.5	2.4		8.7
*V*. *uliginosum*	0.1	1.4	1.5	0.5		2.6
*J*. *communis*	0.0	0.0	0.1	0.0	0.1	
Bioturbation	0.0	0.1	0.7	0.3	0.2	0.0
Erosion/accum.	0.2	2.0	2.7	1.8	0.3	0.3
Fluvial disturbance	**58.5**	6.9	2.8	0.2	4.2	0.5
Solar rad.	0.9	**16.4**	9.8	3.4	0.9	7.8
Litter V	0.7	**21.7**	**24.1**	**16.7**	**32.1**	**29.0**
pH	**16.2**	5.8	9.5	**54.2**	1.2	**19.1**
SWC	1.2	**13.4**	**14.1**	3.5	3.7	9.2
SOM	0.3	**15.2**	**14.5**	9.4	**55.5**	**14.3**
Distance along river	2.1	3.6	6.8	2.2	1.0	3.5
SUM	100.0	100.0	100.0	100.0	100.0	100.0
Type	Species distribution					
Variable	Lichen	Bryophyte	Graminoid	Forb	Dwarf shrub	Shrub
Light attenuation	7.6	3.7	3.8	6.3	3.1	4.9
*E*. *nigrum*	**12.0**	0.7	0.3	0.7		0.5
*V*. *vitis-idaea*	1.9	1.0	0.2	3.6		1.8
*V*. *uliginosum*	1.7	0.3	0.8	1.8		1.0
*J*. *communis*	0.0	0.0	0.0	0.0	0.0	
Bioturbation	0.5	0.2	0.1	0.3	0.4	0.2
Erosion/accum.	1.6	0.7	0.7	1.1	2.0	0.5
Fluvial disturbance	**29.3**	6.5	0.9	0.8	**15.4**	0.8
Solar rad.	3.6	8.5	7.3	7.9	2.6	5.2
Litter V	**16.0**	**57.0**	**41.4**	**27.6**	**20.1**	**33.1**
pH	7.5	2.4	4.8	**11.3**	3.5	**20.8**
SWC	3.0	5.0	**18.0**	8.8	2.3	**13.0**
SOM	8.4	9.6	**12.5**	**18.0**	**45.3**	10.0
Distance along river	6.9	4.5	9.3	11.9	5.4	8.1
SUM	100.0	100.0	100.0	100.0	100.0	100.0
Type	Abundance					
Variable	Lichen	Bryophyte	Graminoid	Forb	Dwarf shrub	Shrub
Light attenuation	7.6	3.5	6.6	6.4	1.8	**(17.2)**
*E*. *nigrum*	**10.8**	2.0	0.4	1.9		0.7
*V*. *vitis-idaea*	4.6	2.8	**10.2**	2.4		2.1
*V*. *uliginosum*	1.1	**16.6**	0.6	1.8		3.4
*J*. *communis*	0.0	0.1	0.0	0.0	0.0	
Bioturbation	0.1	0.3	0.2	0.3	0.3	0.1
Erosion/accum.	0.3	1.0	0.6	2.9	1.3	0.9
Fluvial disturbance	**23.3**	5.4	0.3	2.2	9.1	0.5
Solar rad.	7.3	7.0	2.9	5.4	2.0	3.7
Litter V	**13.4**	**24.5**	**49.0**	**23.6**	**26.0**	**23.6**
pH	**12.7**	2.8	4.0	**28.8**	2.7	**16.7**
SWC	4.4	**11.5**	5.2	5.1	2.6	4.3
SOM	8.1	**16.6**	**17.2**	**14.5**	**50.8**	**18.2**
Distance along river	6.2	5.8	3.0	4.8	3.4	8.5
SUM	100.0	100.0	100.0	100.0	100.0	100.0

Relative influence (%; [75]) of predictor variables in the BRT models for functional group richness, distribution and abundance. The most influential variables (with relative influence > 10%) for each response variable are highlighted with bold font (see Table 7 for their partial effects). Note that shrub abundance is probably the cause of light attenuation, not the other way round. Litter V = litter volume; pH = soil pH; SWC = soil water content; SOM = soil organic matter.

The BRT methodology is well suited for analysing data with multicollinearity issues and performs well in separating the individual effects of collinear predictors ([59]). However, it must be noted that the three most influential site productivity variables, namely litter volume, soil water content and soil organic matter were moderately or strongly correlated (rho between 0.69 and 0.88; S2 Table). Therefore, their individual effects on vegetation may have been difficult to separate even with advanced modelling methods. Moreover, litter volume and soil organic matter were moderately correlated with fluvial disturbance variables (|rho| between 0.51 and 0.69; S2 Table). This multicollinearity may have complicated the separation of the influence of site productivity variables and fluvial disturbance variables.

**Fig 2:**
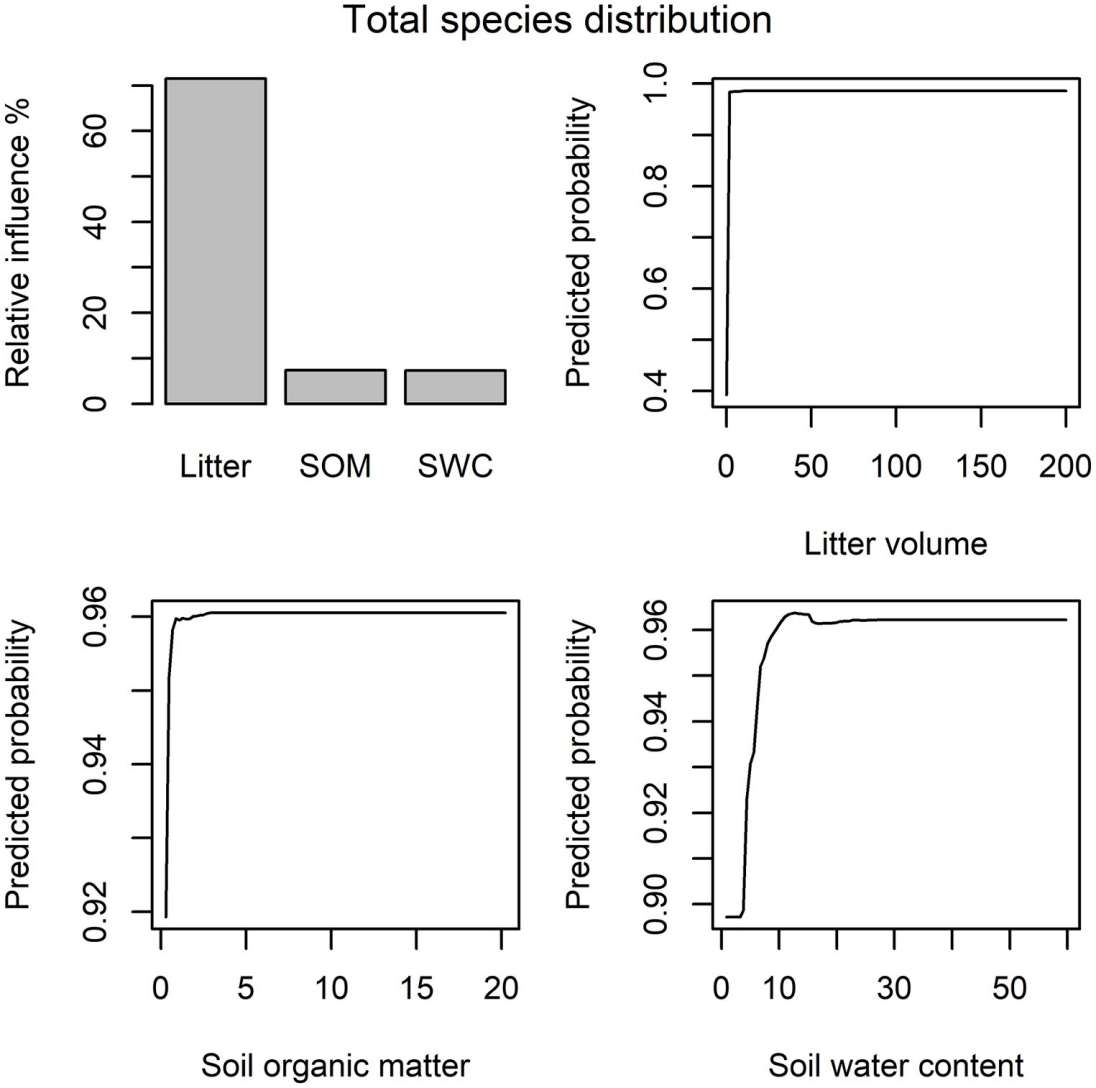
Partial response curves for vegetation cover. Exemplary BRT partial response curves for the most influential (> 7%) predictor variables in the “full” total species distribution model. SOM = soil organic matter, SWC = soil water content.

The effects of litter volume, soil water content and soil organic matter on vegetation variables were mainly positive (Table 7). Across all studied response variables, the curves flattened after a relatively low threshold level was reached (Fig 2). Moreover, their effects on specific response variables were unimodal: for example, forb abundance peaked at intermediate levels of litter volume and decreased slightly when litter volume increased further (Table 7).

**Table 7:** Partial response curves.

Type	Variable	Rel. infl.	Light att.	*E*. *nigrum*	*V*. *vitis*.	*V*. *ulig*.	Fluvial dist.	Solar rad.	Litter V	pH	SWC	SOM
SR	Total	> 7%							+		+	∩
DI	Total	> 7%							+		+	+
AB	Total	> 7%							+			+
SR	Lichen	> 10%		+			─			─		
SR	Bryophyte	> 10%						─	+		+	∩
SR	Graminoid	> 10%							∩		+	∩
SR	Forb	> 10%							+	+		
SR	Dwarf shrub	> 10%							+			+
SR	Shrub	> 10%							+	+		+
DI	Lichen	> 10%		+			─		+			
DI	Bryophyte	> 10%							+			
DI	Graminoid	> 10%							+		+	+
DI	Forb	> 10%							∩	+	+	
DI	Dwarf shrub	> 10%					─		+			+
DI	Shrub	> 10%							+	+	∩	
AB	Lichen	> 10%		∩			─		+	─		
AB	Bryophyte	> 10%				+			+		+	+
AB	Graminoid	> 10%			U				∩			+
AB	Forb	> 10%							∩	+		∩
AB	Dwarf shrub	> 10%							+			+
AB	Shrub	> 10%	(+)						∩	+		+

BRT partial response curves for the 21 response variables in “full” models. Response curves are classified into negative (─), positive (+), unimodal (∩) and U-shaped (U). Note that the positive effect of time since flood on three lichen variables is converted into a negative effect of “fluvial dist.”, since it represents a negative influence of fluvial disturbance. Increase of shrub abundance with increasing light attenuation (in brackets) is probably not caused by decreased light availability. Instead, light availability in the field layer is decreased by increasing shrub abundance. SR = species richness; DI = species distribution; AB = abundance; Light att. = Light attenuation; E. nigrum = *Empetrum nigrum*; V. vitis. = *Vaccinium vitis-idaea*; V. ulig. = *Vaccinium uliginosum*; Fluvial dist. = one of the fluvial disturbance variables; Solar rad. = solar radiation; Litter V = litter volume; pH = soil pH; SWC = soil water content; SOM = soil organic matter.

When examining vegetation as a whole, increasing litter volume increased species richness, probability of vegetation occurrence and vegetation abundance (Table 7; Fig 2). Soil water content had a positive effect on species richness and occurrence probability (Table 7; Fig 2). Vegetation occurrence probability and abundance were positively influenced by soil organic matter, while the effect on species richness was unimodal: species richness peaked at intermediate levels of soil organic matter (Table 7, Fig 2).

**Fig 3:**
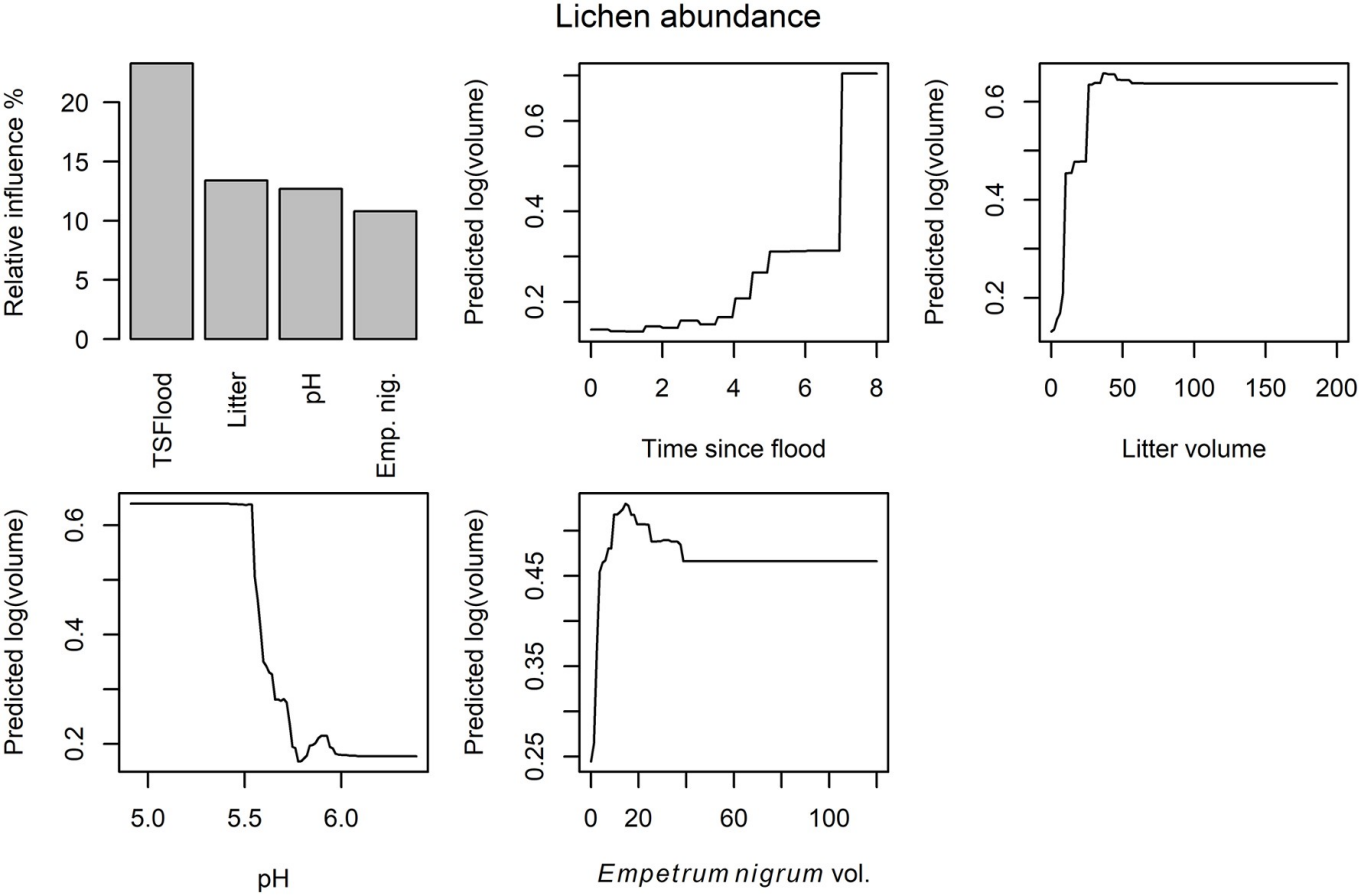
Partial response curves for lichens. Exemplary BRT partial response curves for the most influential (> 10%) predictor variables in the “full” lichen abundance model. TSF = time since flood; E.nig = abundance of *Empetrum nigrum*.

The effect of pH on functional species groups varied: while increasing pH had a positive effect on six response variables, it had a negative effect on lichen species richness and abundance (Table 7). The effect of the fluvial disturbance variable on lichen response variables and dwarf shrub occurrence probability was negative (Table 7; Fig 3; note that effect of “time since flood” on three lichen variables was converted from positive to negative since it represents a negative influence of fluvial disturbance). Solar radiation had a negative effect on bryophyte species richness (Table 7). The abundance of the dominant dwarf shrub species had positive effects on the lichen richness, occurrence probability and bryophyte abundance (Table 7). However, lichen abundance peaked and graminoid abundance reached its minimum at intermediate levels of dominant dwarf shrub abundance (Table 7). Light attenuation was positively associated with shrub abundance (Table 7). However, since this predictor variable measured how much light availability diminished below canopy and shrub layers, the association was probably caused by the shadowing effect of shrubs: when shrubs were present, light attenuation was higher.

### Influence of the components of disturbance and vegetation establishment

Peak stream power either in 2016 or during 2009–2016 was identified as the best fluvial disturbance predictor for most response variables (10 and 8, respectively; S3 Table) and selected for multivariate modelling. The best correlate for total vegetation and graminoid variables varied depending on the type of variable (species richness, distribution or abundance), while one single fluvial disturbance variable was identified as the strongest correlate with other functional groups (S3 Table):

- Lichens–Time since flood
- Bryophytes–Peak stream power during 2009–2016
- Forbs–Peak stream power in 2016
- Dwarf shrubs–Peak stream power during 2009–2016
- Shrubs–Peak stream power in 2016

**Fig 4:**
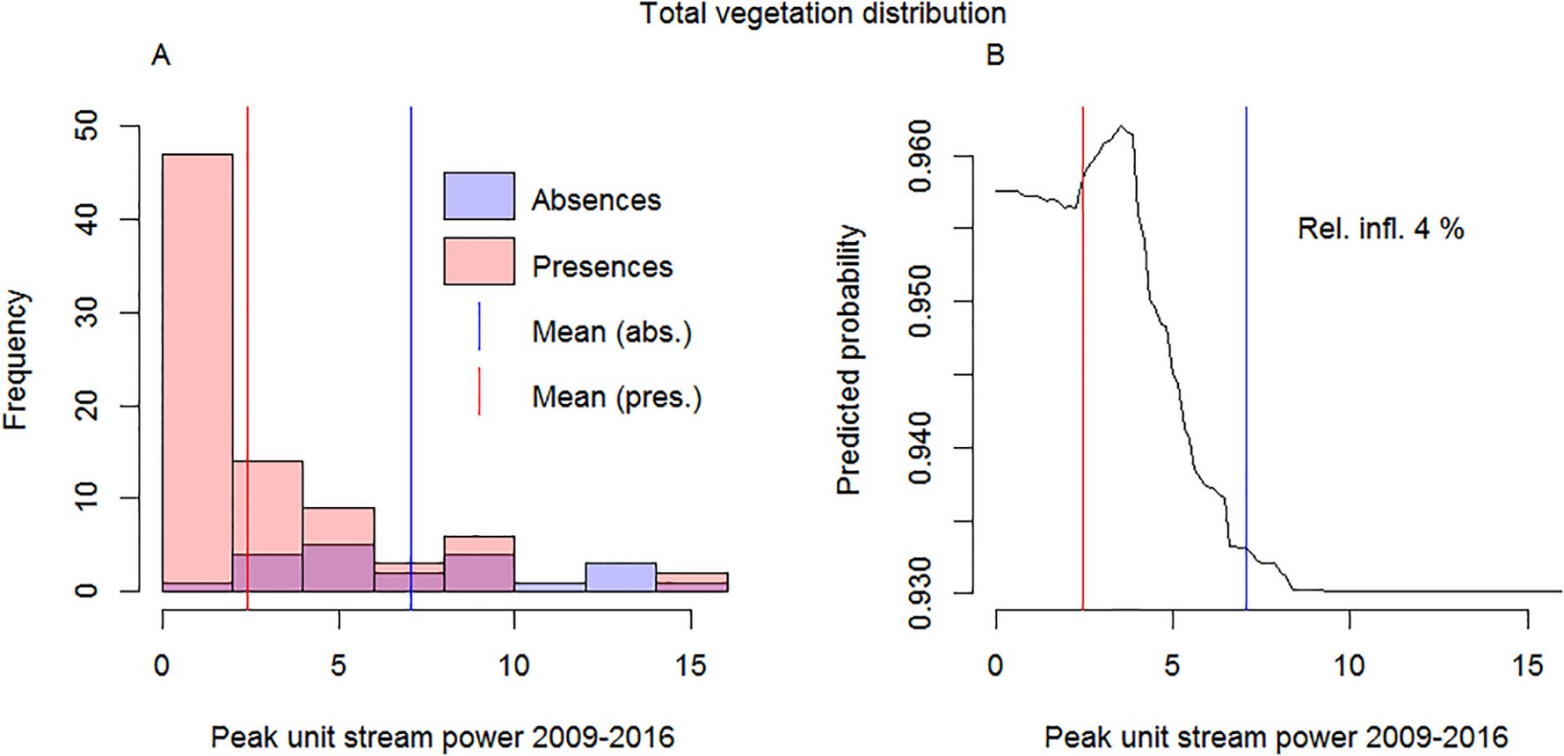
Vegetation along disturbance gradient. The distribution of species presences and absences along the fluvial disturbance gradient (A) and the partial response curve (when the influences of other predictors have been integrated out) for peak unit stream power in 2009–2016 in the total species distribution model (B).

The influence of erosion and accumulation and the fluvial disturbance variables on vegetation variables was relatively low, compared to other explanatory variables (Table 5; Table 6). Vegetation was found along the entire fluvial disturbance gradient, but the probability of vegetation occurrence was negatively influenced by increasing fluvial disturbance. The probability decreased sharply at around 5 W m^-2^ peak unit stream power (Fig 4). While the BRT methodology performs well in identifying the individual effects of collinear predictors ([59]), the moderate collinearity effects between fluvial disturbance variables, litter volume and soil organic matter may have masked some of the influence of the fluvial disturbance variables.

**Fig 5:**
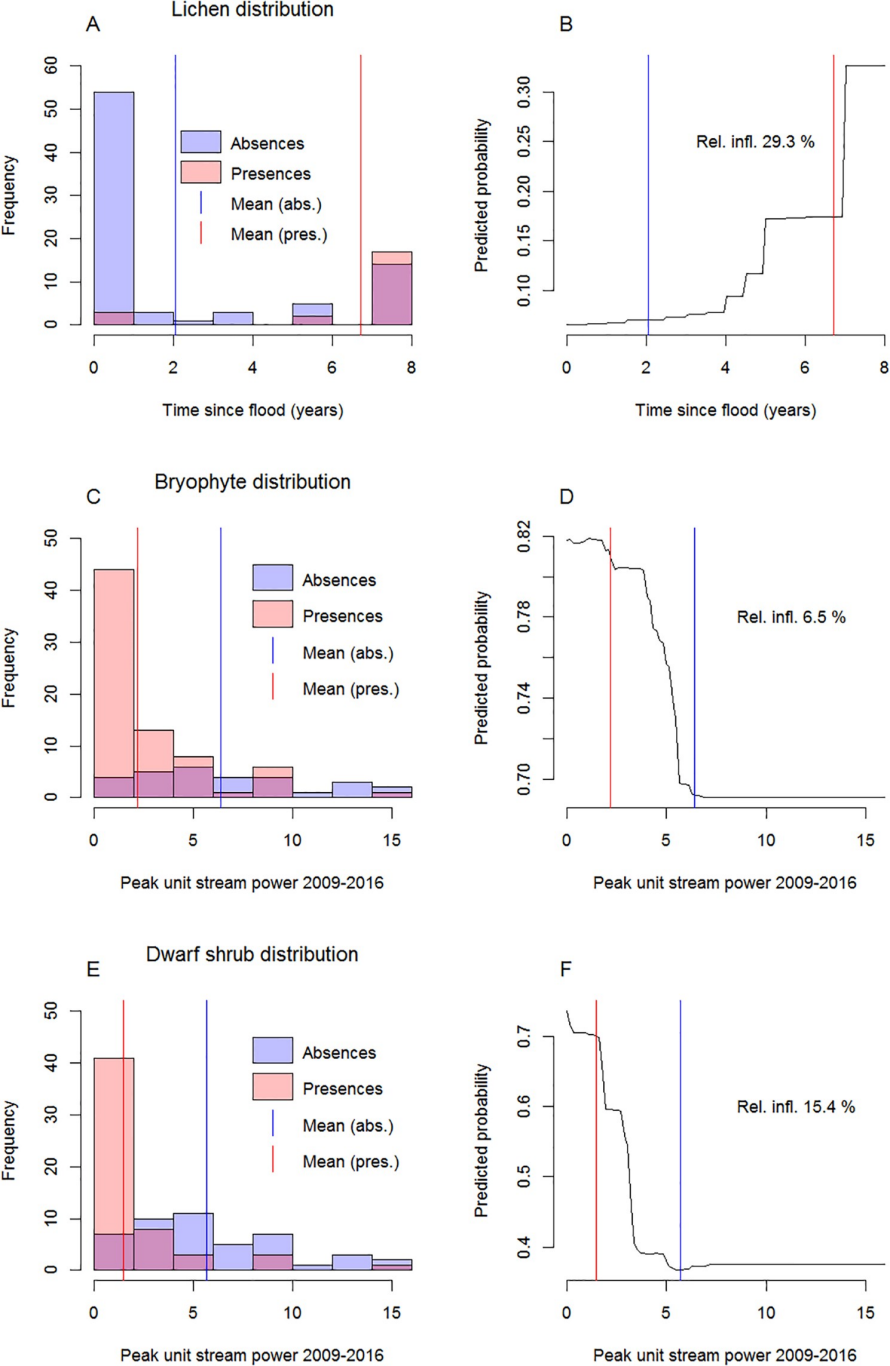
Functional groups along disturbance gradient. The distribution of lichen, bryophyte and dwarf shrub species presences and absences along the fluvial disturbance gradient (A, C, E) and the partial response curve (when the influences of other predictors have been integrated out) for the fluvial disturbance variable in the corresponding species distribution models (B, D, F).

**Fig 6:**
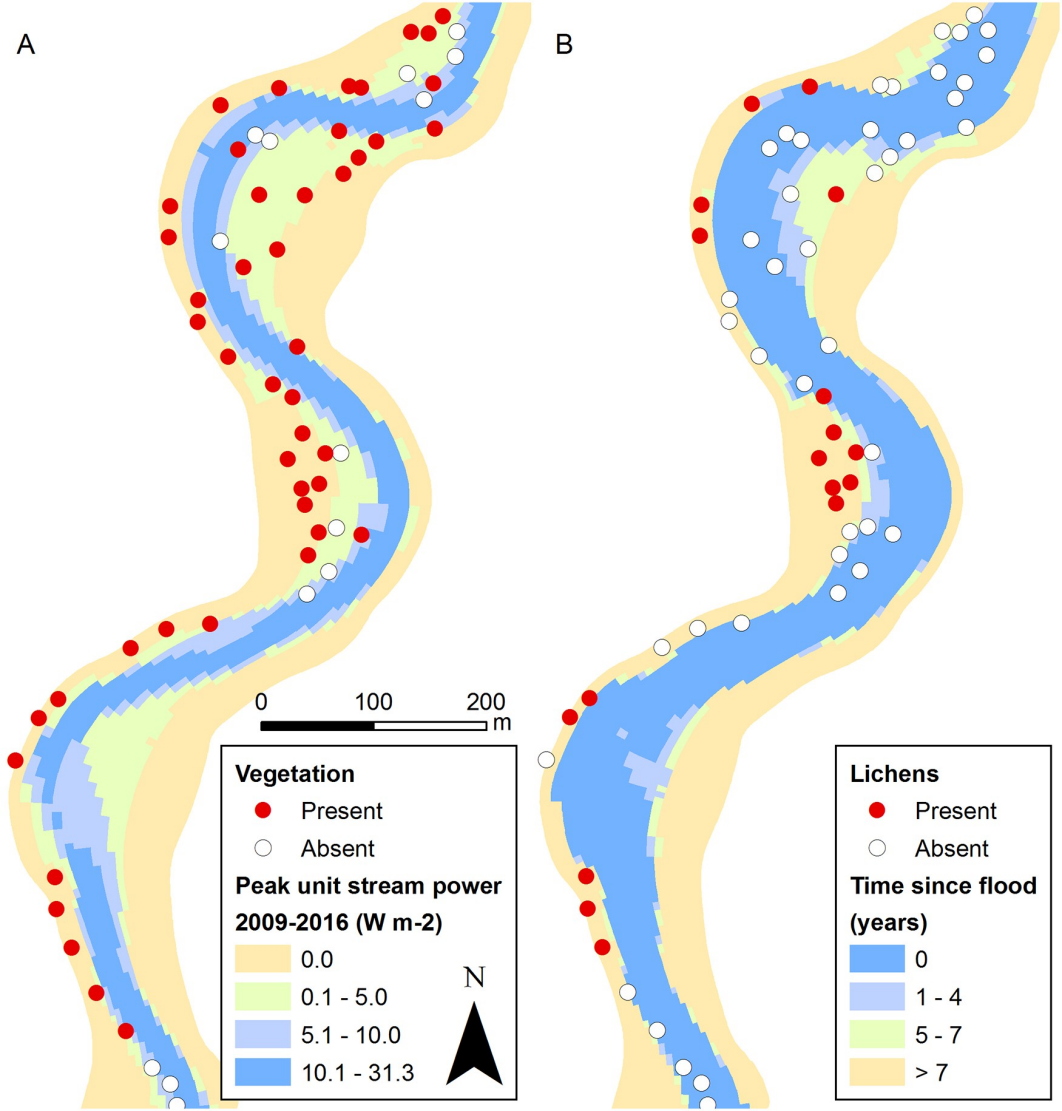
Spatial distribution of vegetation in relation to disturbance. The distribution of vegetation in relation to peak unit stream power (A) and lichen distribution in relation to time since last flood (B). “Full” BRT models predict the probability of vegetation occurrence to increase sharply when peak unit stream power falls below 5 W m^-2^ (green and yellow areas). Lichen occurrence probability is predicted by the “full” BRT model to increase after four years since last flood (green and yellow areas).

The fluvial disturbance variable had a notable relative influence in the lichen, bryophyte and dwarf shrub models, particularly in the species distribution models (Table 6). However, all functional groups occurred along the entire fluvial disturbance gradient (Fig 5). Fluvial disturbance had a negative influence on the occurrence probability of all these functional groups (Fig 5). Bryophyte occurrence probability decreased sharply at around the same disturbance level as that of the entire vegetation community (peak unit stream power c. 5 W m^-2^; Fig 5). Dwarf shrub occurrence probability decreased sharply already at lower disturbance levels (peak unit stream power c. 3 W m^-2^; Fig 5). Lichen occurrence probability was low closely after flood and increased steadily after four years since previous flood (Fig 5). Based on these results, vegetation was expected to occur in large areas for example at the point bars, while the potential areas for lichens were much narrower along the river valley (Fig 6).

## Discussion

The study in a sub-arctic river valley suggests that the complexity of vegetation patterns of the riparian ecosystem can be efficiently regenerated by correlative species richness, distribution and abundance models, when most important factors are incorporated into the models (e.g. [5,6]). The analysis indicates, that the vegetation models may not be significantly improved (nor weakened) when the commonly used proxy variable elevation is substituted with variables based on laser scanning and hydraulic modelling. This may be a local or ecosystem-specific finding: in this data, all these factors (elevation, erosion and accumulation, fluvial disturbance variables, solar radiation) had an overall weak influence on vegetation patterns, when compared to site productivity factors. In this environment, certain vegetation communities grow in areas with a high amount of litter and organic matter in the soil and have existed there undisturbed for many years (probably decades).

In our study area, the moderate multicollinearity effects between fluvial disturbance and site productivity variables may have led to the underestimation of the influence of fluvial disturbance. This may be the case even though the BRT methodology is generally well suited for dealing with collinearity ([59]). However, the incorporation of the biologically more meaningful variables instead of proxies is justifiable in itself ([7,8]) and may facilitate transferring the results into other environments and locations. The transferability into similar environments would be an interesting subject for further studies. In addition, *in situ* measurements of PAR and light attenuation below canopy and soil analyses ensure that most relevant factors are accounted for when for example examining the diversity-disturbance relationship or biotic interactions ([3,4,88]).

Our data illustrates that it is possible to derive multiple biologically relevant variables from mobile laser scanning data and hydraulic modelling. These include solar radiation, peak water depth, peak unit stream power and time since last flood. These variables are more useful than simplistic proxy variables (such as plot elevation that often correlates strongly with vegetation variables; e.g. [89]) in understanding the drivers of vegetation patterns. Moreover, the incorporation of multiple biologically meaningful variables instead of one proxy may bring out some interesting processes and functional group differences ([12]).

Mobile laser scanning is a relatively cost-efficient method for producing precise elevation data and high-resolution DEMs in riparian corridors ([54,55,65]), and can be easily repeated for an estimate of erosion and accumulation along the river banks ([57]). Moreover, when time series of flow conditions are available (at least for the open water periods, which is relatively easy to implement), hydraulic modelling allows the calculation of temporal variation in unit stream power and flood height ([57,58]), which can be summarized into biologically meaningful variables. Unit stream power describes the rate of energy dissipation of water and transported particles against the channel bed (and vegetation) per unit area ([40]). It therefore represents the intensity of disturbance caused by flowing water and particles to vegetation ([41]). Peak water depth indicates if a plot has been inundated by water, which causes e.g. anoxic stress to vegetation ([90]). In addition, water depth influences the severity of the stress ([91]). Estimates of global radiation can be greatly improved by substituting coarse DEMs with laser scanning-based high-resolution DEMs (c.f. [61]).

The results indicate that litter abundance is the most important driver of vegetation patterns in this study setting ([92]). This highlights the role of successional processes ([93]) at this spatial scale in a riparian environment. Based on the results, litter has a uniformly positive effect on species richness, species occurrence probability and abundance in this sub-arctic ecosystem. The effect may be opposite or more complex and species-specific in other (more productive) environments ([34,92]). In the river valley, litter accumulates in areas with existing vegetation cover (thus, vegetation patterns influence the spatial patterns in litter volume, not only vice versa). Slope and fluvial processes redistribute litter also on barren surfaces, facilitating the establishment of species there ([34,92,93]). Since both disturbance and existing vegetation influence the distribution of litter, the effects of litter and disturbance as well as the cause and effect in the litter-vegetation-relationship may be difficult to separate. In our data, the statistical association between litter and fluvial disturbance was not particularly strong (Rho |0.12|-|0.69| depending on the disturbance variable). In addition to litter, soil organic matter and soil moisture are highly influential for the maintenance of vegetation patterns in this study setting.

Expectedly, increasing fluvial disturbance decreases the chances of vegetation establishment in this environment. When examining functional groups separately, this limiting pattern is clear for lichens, bryophytes and dwarf shrubs. This finding potentially reflects the sensitivity of these functional groups to flooding and scouring ([50–52]). Intense fluvial disturbance would presumably limit vegetation establishment across all functional groups, but site productivity is the limiting factor in the sub-arctic river valley, and masks the effect of fluvial disturbance. Species richness and vegetation abundance decrease notably along the fluvial disturbance gradient, but vegetation can be found along the entire fluvial disturbance gradient (above minimum shoreline level). Thus, we cannot determine an absolute disturbance threshold that limits vegetation establishment. This is potentially caused by the presence of (few and scarce) extreme specialist species that tolerate high disturbance conditions (e.g. [12]). In addition, small favourable patches with, for example, high amount of litter and optimal soil moisture conditions may facilitate the establishment of vegetation in otherwise unfavourable disturbance conditions (e.g. [12,93]). This highlights the importance of interaction between site productivity factors and disturbance in such extreme environments.

As we expect (e.g. [12,33,37]), the intensity of disturbance and the duration of the flood-free period are both influential in determining vegetation patterns in this kind of riparian environment. Our results suggest that the length of the flood-free period is more important than the intensity of the fluvial disturbance for lichens, while the intensity is more influential for other functional groups. This difference may also be attributable to the sensitivity of lichen group to flooding (c.f. [50–52]). The results suggest, that erosion and accumulation along the shoreline and slope processes along the river banks are not influential for vegetation patterns, at least at the examined spatial and temporal scales. We hypothesize that this is due to the sudden and localised nature of mass movements along the slopes and the gradual changes in the erosion and accumulation patterns along the channel.

What remains to be tested in future studies, laser scanning data and airborne photography could be even more fully utilised in improving vegetation models. For example, laser scanning could be used in defining the friction parametrization of the hydraulic model ([94]). In addition, combining airborne laser scanning data or photogrammetry point clouds with the mobile laser scanning data has potential in expanding the DEM seamlessly to the river banks ([95]). Digital surface models, including the vegetation, could be generated from multi-source laser scanning data for the river banks ([96,97]), which in turn could be used for taking into account the shading effect of the canopy in densely vegetated areas ([98]). Vegetation metrics and even species distribution data could be determined from suitable remotely sensed data (e.g. [97,98]). Measuring the river geometry each year would also enable analysing annual erosion and accumulation ([55]), and it could be used as a changing hydraulic model geometry to enable more accurate hydraulic modelling.

As previous literature shows, temperature influences vegetation patterns at the landscape scale ([25,26,99,100]). However, the microclimatic variability in a river valley is more unpredictable and difficult to infer from elevation models. Thus, the influence of temperature on vegetation patterns at this scale remains beyond the scope of this study. The incorporation of direct measurements of the microscale variability of surface and topsoil temperature conditions could improve local-scale vegetation models even further.

## Conclusions

Correlative vegetation models are a useful tool for examining the drivers of vegetation patterns. Robust inference requires trying to incorporate all important factors in the models and using biologically meaningful variables to quantify them. We demonstrate how *in situ* measurements, mobile laser scanning and hydraulic modelling can be combined to quantify the key environmental patterns. Our results from a sub-arctic river valley indicate that the correlative vegetation (vascular plant, bryophyte and lichen) models built on these environmental variables succeed in predicting riparian vegetation patterns and highlighting differences between functional species groups.

Our results are in line with the common finding that elevation is a useful measure of relative position along the main local environmental gradient and an effective predictor variable in vegetation models. However, using directly measured and modelled variables allow relating vegetation patterns e.g. to stream power or the length of the flood-free period. Substituting a “practical proxy variable” with biologically relevant variables in correlative vegetation models raise important processes and may allow more precise between-site comparisons.

The amount of litter is the dominant driver of local-scale variation of species richness, distribution and abundance across all functional groups in this sub-arctic riparian environment. In addition, soil organic matter and soil water content are important factors influencing vegetation patterns. Fluvial disturbance is a key limiting factor only for lichen, bryophyte and dwarf shrub species. The long-term peak intensity of stream power is the most important component of disturbance for most functional groups, while the length of the disturbance-free period is more relevant for lichens.

In conclusion, seeking biologically meaningful quantifications of environmental drivers may bring out important processes and functional group differences related to vegetation patterns. Mobile laser scanning, high-resolution DEMs and hydraulic modelling offer valuable solutions for improving correlative vegetation models. Thus, they allow us to better examine the vulnerability of vegetation to environmental change and to project future vegetation patterns.

## Supporting information

S1 Table Input data for the analyses. The field survey site is located in the lower Pulmanki River (N 69° 55.111', E 028° 01.664'; N 69° 56.281', E 028° 02.631').
(XLSX)

S2 Table Spearman rank correlations of potential predictor variables. High correlations (|rho| ≥ 0.7) and highlighted with bold font.
(XLSX)

S3 Table Spearman rank correlation between response variables and fluvial disturbance variables. The highest value (and thus the fluvial disturbance variable selected for multivariate modelling for each response variable) is highlighted with grey background colour. Peak water depth in 2016 and in 2009–2016 were not selected for any models and are therefore not shown in this table.
(XLSX)

S4 Table Moran’s I statistic and associated p-value for raw values and BRT model residuals. The reduction in the significant spatial autocorrelation is shown before and after the inclusion of the dummy variable “distance along river”. Significant (p < 0.01) values are highlighted with grey background colour.
(XLSX)
